# Predictors of happiness among retired from urban and rural areas in Brazil

**DOI:** 10.1186/s41155-016-0055-3

**Published:** 2017-01-19

**Authors:** Silvia Miranda Amorim, Lucia Helena de F. P. França, Felipe Valentini

**Affiliations:** Graduate School of Psychology - University Salgado de Oliveira - UNIVERSO, Niterói, Rio de Janeiro, Brazil

**Keywords:** Retirement, Happiness, Rural area, Urban area

## Abstract

This study compared differences in degree of happiness, social support, activities performed, and health and economic situation among retirees from urban and rural areas in Minas Gerais State in Brazil. The influences of these predictors over individuals’ level of happiness were also analyzed.

We included 279 retired individuals living in Abre Campo (a municipality with a population fewer than 20,000 inhabitants, which is considered a rural area) and in Belo Horizonte (a municipality with a population of almost 2.5 million inhabitants, which is considered an urban area). Participants responded to a questionnaire that included scales of happiness, social support, diversity of activities, and issues about satisfaction with health and economic situation. Retirees from the urban area had a higher happiness level than retirees from the rural area (*β* = 0.16). The most important predictors of happiness were health (*β* = 0.42), social support (*β* = 0.26), and economic situation (*β* = 0.15), but no moderation effects of urban and rural areas were found. Our findings support the implementation of actions to offer financial planning before retirement and to stimulate social support and health promotion for retirees, particularly given the importance of these factors in perception of happiness.

## Background

The interest of psychologists in the characteristics and positive experiences of life—such as happiness—developed in the beginning of the twentieth century with the positive psychology movement (Seligman 2002). Csikszentmihalyi and Hunter (2014) claimed that a good level of happiness is achieved when a person experiences a high degree of positivity or pleasant affect, such as joy, pride, and satisfaction with his/her own life, as well as a low degree of negativity or unpleasant affect, such as sadness, depression, and envy. Therefore, a happy person is not one who does not experience depression but someone who has experienced a good number of emotions and has positive cognitive status with regard to different aspects of life (Lyubomirsky et al. 2005).

Lyubomirsky and Lepper (1999) established that “subjective happiness” is a person’s assessment of himself or herself being happy or unhappy. Lyubomirsky (2001) highlighted on how impressive is the ability of some individuals to be happy even under adverse circumstances, although natural or learned factors that explain this ability are unclear. Seligman (2002) believes that among external circumstances influencing feelings of happiness are income, marital status, social life, positive emotions, age, and health.

In retirement seen through positive psychology, happiness is related to the concept of successful aging (Adams and Taylor 2015). The leaving of the working environment entails changes in daily routine that determine important changes in the life cycle and contributes to retirees’ need to define themselves in ways not related only to work (Van Solinge 2013; Van Solinge and Henkens 2008). Furthermore, the changes in lifestyle for people to be retired is devalued by society and sometimes equated to uselessness. This can occur when retirees have placed the work in the central position of their life compared to other aspects, turning the end of working into a negative experience (Gallo 2013; França 2012). On the other hand, retirement can be seen as an advantage or gain, especially in terms of additional time available for performing and developing other projects and adopting a new lifestyle (França 2012).

For these reasons, retirement has been considered a major transition in the life of adults, and if they do not plan for it early, this experience can become traumatic (Van Solinge and Henkens 2008). The factors relevant for planning retirement are (i) long-term risk factors, such as health promotion (Kubicek et al. 2011; Van Solinge and Henkens 2008), financial aspects (Hershey et al. 2010), and individual and collective quality of life (França 2004; Peiró et al. 2013); (ii) medium- and short-term factors of well-being, such as intellectual development and diverse activities (social and leisure activity, hobbies, cultural and physical activities, volunteer opportunities, and routine tasks) (Nimrod and Shrira 2014; Oerlemans et al. 2011); and (iii) life-long learning (França 2012), seeking social support (Wang and Schultz 2010), and work, social, affective, and family bonds (Antonucci 2001). Brazilian studies on key aspects of retirement planning have found that for both executives (França 2004) and non-management workers, the most important factor in life is health, especially aspects related to nutrition. Other important factors are social and family relationships and diversity in leisure, volunteer, and household activities.

Few of the factors described above have been discussed in the literature so far, especially well-being and happiness during retirement and possible differences among people living in urban and rural areas. However, first studies that compared welfare both in rural or urban areas are dated to the 1980s when Kozma and Stones (1983) and Flenger and Jensen (1981) investigated differences in happiness and life satisfaction between urban and non-urban population.

These studies did not report a consensus on which area people would be happier. Some authors have concluded that urban environment would be more favorable to life satisfaction of elderly people (Flenger and Jensen 1981), but others strongly endorse that the rural environment seems to be a better predictor of happiness (Kozma and Stones 1983). They also confirmed the importance of resources such as healthcare, social and physical activities, and community support (Kozma and Stones 1983) and the differences given between the two environments (Flenger and Jensen 1981). These results were reinforced by the subsequent study by Mongilner (2009) that compared individuals from rural and urban areas, suggesting the great importance given to social relationship was associated with lower importance attributed to personal gains, and economic status would result in higher happiness level.

Cohen and Bulanda (2015) indicated the social aspect as one of biggest benefits of aging in rural areas because life in the community becomes more intimate. This, in turn, strengthens bonds and reduces the risk of becoming anonymous or forgotten. Santos et al. (2013) identified other positive characteristics of rural areas, such as slow life rhythm, which involves lower pressure, a high feeling of safety, and large consonance with the area, which generally facilitate the activities of caring for animals and contact with nature.

On the other hand, municipalities that have recently acquired urban characteristics in general offer greater resources and provide more reasons for successful aging, and such characteristics may minimize worsening of disparities (Silva and Welgama 2014). Furthermore, in urban areas, the population has, in general, higher level of education and income, which can contribute to the decision to retire, can facilitate the possibility of a new beginning during retirement, and can directly influence health condition and quality of life (Santos et al. 2013).

There is still an idea of difference between rural and urban areas related with development level of each country. Requena (2015) found that poor infrastructure in rural areas, especially in underdeveloped countries, is associated with a lower self-evaluation of welfare. However, at developed countries, in which satisfaction with infrastructure is high, people living in rural areas seems to be more satisfied than those living in urban areas.

People share the worry of finding a friendly environment that is not excessive in some way, is not lacking basic necessities, and that allows interventions to enable solutions for difficulties of vulnerable populations (Navarro et al. 2015; Ruza et al. 2015). For this reason, it is relevant to study the influence of specific contexts in elderly people’s well-being; from this analysis arises an understanding about residents and assistance for their needs. Based on these studies, there is still further investigation on living conditions in rural and urban areas that could help a future theoretical framework, which still requires study: does the area—urban or rural—in which retirees live help or harm their feelings of happiness? And, how is the difference of retirees’ perception from both areas?

### Present investigation

To improve understanding of retirees’ lives in urban and rural areas, we investigated the perception of happiness and its predictors among retirees from two municipalities, including urban and rural areas. We also sought to evaluate the difference in social support received and activities performed by the two groups—rural and urban. We called rural area to define the locality that has less than 20,000 inhabitants and the urban area as the locality that has more than 20,000 inhabitants (Martins et al. 2007). In our research, we choose Abre Campo as the rural area and Belo Horizonte as the urban area, both cities are located in the State of Minas Gerais, Brazil.

The literature review described above enabled the development of three hypotheses: (i) the more diverse the activities engaged in by retirees, the greater their subjective happiness will be; (ii) satisfaction with one’s financial situation will be positively related to respondents’ perception of happiness, independently of the region in which one lives; and (iii) retirees living in rural areas, such as Abre Campo, have more resources for social support compared to individuals living in a more urban environment, such as Belo Horizonte, which in turn will have a positive influence on their perceptions of happiness. These three hypotheses were tested by estimating series of structural equation models in which a measure of subjective happiness represent the criterion variable. Predictors were measurement of living situation (rural/urban), social support, financial situation, and the frequency of activities (social, physical, routine, leisure).

## Methods

### Participants

Municipalities were selected by using the Brazilian Census of 2010 (IBGE 2012). We selected those with less 20,000 inhabitants, which were considered rural municipalities, and those with more than 20,000 inhabitants, which were considered urban areas. Among these municipalities, we chose, by convenience, two cities in Minas Gerais State: Abre Campo (rural municipality) and Belo Horizonte (urban municipality). We included participants who were retired and lived in one of these two municipalities.

Data collection resulted in 279 completed questionnaires (48% from retirees living in the urban area and 52% from retirees living in the rural area); more than half of participants were female (60.0% vs. 39.4% male). Participants were aged 49 to 91 years, and the mean age was 68.7 years (SD = 9.2 years). Concerning marital status, little more than half of retirees (56.6%) were married or cohabitants, a little more than one fourth (27.3%) were divorced or widowed, and one tenth had never married or been cohabitant. The situation was similar for housing: More than half of participants (52.6%) lived with a partner, one third lived with relatives and friends (29.4%), and one tenth lived alone. Concerning formal education level, most participants had completed middle-school (36.5%), little more than one fourth had completed high school (27.6%), and 14.4% of them finished undergraduate or graduate studies. However, only 16.7% of participants were literate.

### Instruments

#### Dependent or criteria variable—subjective happiness scale

The subjective happiness scale was created by Lyubomirsky and Lepper (1999) and validated in Brazilian Portuguese by País-Ribeiro (2012). The instrument uses a Likert scale with four anchoring points (raging from 1 [less happy] to 7 [more happy]). In the Brazilian validation conducted by Damásio et al. (2014), the test for hypothesis that four items comprise a single factor showed good adjustment. Therefore, the authors believe that the Brazilian version of the subjective happiness scale presents identical psychometric properties of the original version. In the validation study with retirees from Minas Gerais State (Amorim 2015), the scale presented one dimension of four items, which explained 59.44% of variance. Correlations among items ranged between 0.50 and 0.94, and the internal consistency was satisfactory (*α* = 0.76).

#### Social support scale

The social support scale—created by Sherbourne and Stewart (1991) and validated in Brazil by Griep et al. (2005)—has 19 Likert-type items, ranging from 1 (never) to 5 (always), covering affective, emotional, and material dimensions. In a validation study for retirees of Minas Gerais State (Amorim 2015), the scale was presented as one-dimensional; it kept the 19 items and explained 74.30% of variance. Correlations among items ranged from 0.83 to 0.92, achieving an excellent internal consistency (*α* = 0.98).

#### Diversity of activities scale

Diversity of activities index or sum of diversity of activities (SOD) was developed by França (2004) in a survey administered to Brazilian and New Zealanders executives, and this measure assesses the attitudes regarding retirement. Adaptation and validation of SOD, currently named scale of diversity of activities (SDA) for retirees of Minas Gerais State (Amorim 2015), resulted in a Likert-type scale ranging from 1 (never) to 5 (always) with 24 items. Indicators of internal consistency ranged from 0.73 to 0.84.

We also applied an instrument with socio-demographic questions: age, sex, level of education, marital status, and residency situation. The instrument contained two additional questions on the economic satisfaction of retirement (What is your level of satisfaction with your income today? Score ranged from 1, very dissatisfied, to 5, very satisfied) and health self-perception (How do you rate your health today? Score ranged from 1, requiring care, to 5, excellent).

### Procedures

The project was approved by the Ethical and Research Committee of the University. Participants revised and signed the consent form, which guaranteed confidentiality of information and anonymity.

Convenience sampling procedures were used, selecting accessible individuals who satisfy the inclusions criteria. The data were collected in churches, public spaces, retirement associations, downtown areas, public parks, healthcare facilities, and retirement events, where the retirees were invited to participate in the study by answering a questionnaire. The inclusion criteria of the participants were to be retired and to reside in one of the two selected cities. The participants answered a questionnaire by themselves, receiving assistance when they needed.

Data were analyzed using conventional structural equation modeling techniques. All scales used were configured as latent variables in models. To facilitate estimation of parameters, items of social support scale were grouped in three parcel items on the basis of covariances between items and different intercepts. Parameters of items were estimated by maximum likelihood with standardized errors (MLF), implemented in Mplus software (v. 7.11), and integration algorithm (Monte Carlo). We also separated tested models by urban and rural groups, which were fixed with items’ parameters (loadings and intercepts) as being equal across groups. This procedure was performed using a multi-group structural equation modeling approach, which aimed to guarantee that possible differences in regression coefficients between rural and urban groups could be attributed to group status instead of differences among parameters of the items.

## Results

### Measurement model

To create parcel items, we estimated models of confirmatory factor analyses for each scale used in this study. Items were grouped according to covariances and intercepts in a manner in which parcels included different levels of endorsement probability.

On the basis of parcel items, we estimated a unique measurement model by setting all latent variables simultaneously. This procedure aimed to evaluate discrimination among factors. This model showed acceptable goodness of fit [*χ* ^2^(df) = 131.61 (67); TLI = 0.96; CFI = 0.97; RMSEA = 0.06].

To amplify the analysis of the measurement model, we presented factor loadings, the average variance extracted (AVE), and correlations among latent variables (Table 1). For scales, loadings were equal to or greater than 0.60, with the exception of three items on the happiness scale, and the frequency and diversity on the activities scales. AVE also indicated that latent variables explained, on average, more than 45% of variance of the items. Concerning relations among variables, we found that AVE values were higher with determination coefficients (*r* ^2^) among latent variables (i.e., AVE >*r* ^2^), which indicates lack of multicollinearity. These findings also provided evidence that the latent variables included in the study showed internal structural validity.

**Table 1:** Correlations between study variables and average variance extracted (AVE)

	VME	1	2	3	4	5
1. Subjective happiness	0.48		*0.11*	*0.01*	*0.07*	*0.22*
2. Social support	0.87	0.33		*0.01*	*0.02*	*0.03*
3. SDA	0.45	−0.03^ns^	−0.02^ns^		*0.03*	*0.03*
4. Assessment of economic situation	^a^	0.26	0.15	0.16		*0.10*
5. Health assessment	^a^	0.47	0.17	0.16	0.32	

*Note:* The inferior diagonal presents correlations among latent variables, estimated by structural equation models; the superior diagonal presents coefficients of determination (i.e., squared correlations); all correlations above 0.06 were statistically significant (i.e., *p* < 0.05, if *r* ≥ ±0.06)

*SDA* scale of diversity of activities, *ns* non-significant

^a^Variable measured by a single item

### Explicative latent models of subjective happiness

A structural equation model (SEM) was used to evaluate the model of relation among variables of the study. Such a hypothetical model sets the variables of social support, economic situation assessment, health assessment, and frequency of activities as explanation for the variable of subjective happiness. In addition, the hypotheses of the model suggest that these relations would be moderated by rural and urban areas.

The first model (Table 2) presents goodness of fit index lower than what is expected [*χ* ^2^(df) = 251.37 (81) add *p* level here; TLI = 0.86; CFI = 0.89; RMSEA = 0.08], and variables explain 30.4% of the variance in happiness scores. In the total sample, the health assessment showed an effect on subjective happiness. On the other hand, the variables sex and frequency and diversity of activities did not present significant influences on the dependent variable of subjective happiness. Therefore, they were removed from subsequent models.

**Table 2:** Structural equation models with fixed effects examining predictors of happiness

Effects	Model 1		Model 2	
	*B* (SE)	*Beta*	*B* (SE)	*Beta*
Social Support → Happiness	0.23 (0.07)*	0.25	0.23 (0.07)*	0.26
Economic S → Happiness	0.16 (0.07)*	0.15	0.15 (0.07)*	0.15
HealthA → Happiness	0.40 (0.09)*	0.44	0.38 (0.09)*	0.42
Group → Happiness	0.33 (0.12)*	0.16	0.35 (0.12)*	0.18
Sex → Happiness	−0.17 (0.13)^ns^	−0.08		
SDA → Happiness	−0.07 (0.06)^ns^	−0.08		

*Notes:* Happiness (latent variable); Social support (latent variable); Group (1 = rural; 2 = urban); Sex (1 = women; 2 = men)

*Economic S* assessment of economic situation, *HealthA* health assessment, *SDA* scale of diversity of activities, *ns* non-significant (*p* > 0.05)

*p < 0.05

The second model removed variables that did not present statistical significance in the first model. Therefore, the second model had acceptable adjustment of data [*χ* ^2^(df) = 95.71 (31); TLI = 0.91; CFI = 0.93; RMSEA = 0.07], and the variables social support, economic situation assessment, health assessment, and group (rural or urban) explained 28% of variance of happiness. Such results indicate that, even after removal of the variables sex and frequency of activities, the reduction of explanation of variance is small. Thus, the second model is more parsimonious.

After that, a model was tested (not reported in tables), which considered the group (rural or urban) as a moderator of the effects between subjective happiness and independent variables (social support, economic situation assessment, and health assessment). For this reason, the regression parameters were randomized, which implies the parameters were set to freely vary among participants. However, this model showed a non-positive defined matrix. In this model, parameters of random regression for health and economic situation assessment had variance equal to 0. Therefore, these parameters were configured as fixed in the subsequent model. Model 3 was configured with a direct random effect and two fixed effects (Table 3).

**Table 3:** Structural equation models predicting happiness with random effects and fixed effects according to group (rural/urban)

Effects	Model 3	Model 4			
	Fixed and random slopes	Fixed items parameters (multi-group) and free regression coefficients for groups			
		Rural (*n* = 145)		Urban (*n* = 134)	
	*B*	*B*	*Beta*	*B*	*Beta*
P1: Social support → Happiness	0.27 (0.11)*^,a^ interc.	0.32 (0.09)*^,b^	0.38	0.16 (0.07) *^,b^	0.18
EconomicS → Happiness	0.10 (0.07)^ns^	0.20 (0.09)*^,b^	0.20	0.05 (0.12)^ns,b^	0.04
HealthA → Happiness	0.36 (0.08)*^,b^	0.28 (0.08)*^,b^	0.35	0.45 (0.13)*^,b^	0.44
Group → P1	−0.07 (0.14)^ns^				

*Note:* Variables of social support and happiness were configured as latent. Group (1 = rural; 2 = urban)

*EconomicS* assessment of economic situation, *HealthA* health assessment, *interc.* intercept of a random effect, *P1* random parameter/effect 1, *ns* non-significant (*p* > 0.05)

*p < 0.05

^a^Random effect

^b^Fixed effect

Model 3 shows that the fixed effect was statistically significant between health assessment and happiness and the fixed effect was not significant between economic situation and happiness. Moreover, the random positive effect between economic situation and happiness was not significantly moderated by group (rural or urban).

Considering that the random effect model (model 3) did not present significant moderation, and because of difficulties with identification and non-positive matrixes, we sought to evaluate an alternative model with fixed effects separated by rural and urban groups (model 4). Because of the configuration of happiness and social support as latent variables, differences among the item parameters could yield, hypothetically, different estimation of the regressions coefficients between rural and urban groups. To overcome this problem, in model 4, we fixed the factorial structures, loadings, and intercepts as equal for rural and urban groups. Therefore, we guaranteed that the differences in effects of regressions between groups were not biased by latent variable structures.

Table 3 shows that effects of economic situation on happiness are statistically significant for the rural group, but not for the urban group. Effects of social support and health assessment on happiness were statistically significant for both groups. However, differences between regression coefficients (effects) of rural and urban groups were not statistically significant (*p* > 0.05). The model for the rural group explained 33.8% of the variance of happiness, and the model for the urban group explained 23.7%.

## Discussion

To answer the initial question of this study about the differences and similarities in the happiness level among retirees living in urban and rural areas, we investigated 279 retirees of two municipalities of Minas Gerais: Belo Horizonte (almost 2.5 million inhabitants) and Abre Campo (fewer than 20,000 inhabitants). One hypothesis suggested that the more diverse the activities engaged in by retirees, either in the urban or the rural zone, the greater would be their happiness. We also expected that retirees’ current financial situation would influence the perception of happiness and that residents of Abre Campo would have more resources of social support, but there were no differences in their perceptions about happiness.

The initial model for both groups showed that subjective happiness would be explained by the variables social support, frequency and diversity of activities, economic situation assessment, health assessment, group (rural or urban area), and sex. We did not observe a significant relationship between subjective happiness and activities done by retirees, refuting our first hypothesis. França (2004), in a study of 517 Brazilian and New Zealander executives, reported that diversity of activities was one of the largest predictors of positive attitude in retirement, especially for Brazilian executives and the oldest individuals who participated in the study. This was reinforced by Nimrod and Shrira (2014) concluded that leisure activities become more important for people as they age.

In our research, we did not find any significant relationship between the diversity of activities and happiness. Further studies are needed to replicate this scale in other groups in order to confirm the importance of this predictor on happiness for Brazilian retirees.

On the other hand, subjective happiness was explained by social support, economic situation assessment, health assessment, and area of residence. Social bonds were considered important for health maintenance and coping with several situations, as previously reported by Antonucci (2001), Griep et al. (2005), and Van Solinge (2013). The importance given to economic situations and health are expected results, based on the literature. The daily routine of individuals requires a basic plan to obtain financial balance and avoid habits or lifestyles that compromise their health, as well as actions that promote physical, emotional, and social integrity, causing to some extent the happiness status (Heybroek et al. 2015).

We confirmed our hypothesis that the greater the satisfaction with one’s financial situation, the higher would be the subjective happiness index. These results corroborate with the conclusions of Van Solinge (2013) who believed that good financial planning can lead to the anticipation of resources that facilitate the adjustment for retirement. Authors such as Hershey et al. (2010) showed how financial resources and planning could help maintain an adequate and healthy lifestyle after retirement. França (2012) believes that financial resources are one of the risk predictors of well-being in retirement and, therefore, older persons should be planning at least 2 years prior the transition to retirement.

In a study model, the area where an individual lives was not a moderator of the relationship between happiness and independent variables, and when retirees of each area were considered separately, no significant differences were seen. Therefore, our last hypothesis, about social support being higher in rural areas, was not confirmed. Other Brazilian studies carried out in the Northeast region of Brazil did not find a significant difference in quality of life between rural and urban areas (Martins et al. 2007).

Despite this fact, we observed that retirees in the urban area had higher levels of subjective happiness than those who lived in the rural area. This result would be related not to the area in which the retiree lived, especially because the area was not a moderator among variables, but to a combination between importance attributed to social aspect and lower importance to financial aspect (Mongilner 2009).

In general, the results of this study highlighted the importance of proximity of friends, family, and partners in the life of retirees in the two municipalities of Minas Gerais where this study was conducted, as well as health and finance aspects. The financial situation explained part of the subjective happiness of retirees, which reinforced the need for a planning strategy in this area.

In addition to these results, the reflection about the demographic transition process that we are going through can encourage the adoption of some strategies by the government and professionals seeking to promote happiness for retirees. Besides that, we believe that there should be more social centers for retirees to increase opportunities for social relationships and friendship (Antonucci 2001), long learning center for those who are unemployed or need to updated their knowledge to return to the labor market (Wang and Shultz 2010), as well as retirement support groups for those who plan to create new life projects. We believe we have achieved our goal on discussing the main issues related to resources available in large and small cities, which contribute to the level of happiness of the inhabitants of these spaces.

Large cities can offer more facilities to their inhabitants, a variety of activities, and access to health services and the infrastructure needed for their citizens to age well and healthy. Small cities have an advantage because they are more nurturing, and this can transform or neutralize the feeling of loneliness (Santos et al. 2013; Cohen and Bulanda 2015). However, this kind of accessibilities is harder in the rural area, which can increase the risk factors and prevent the opportunity for a new beginning that retirees often envision (Santos et al. 2013; Silva and Welgama 2014).

This study has, at least, three limitations to be cited. The first is related to the study’s focus on only two cities in Minas Gerais State, making it impossible to generalize the results to the whole country of Brazil. The second limitation is that happiness of retirees could be related to other personal issues, such as optimism, resiliency, and personal characteristics that were not tested, according to Lyubomirsky (2001) and Kubicek, et al. (2011). These limitations led to recommendations for future studies involving these personal qualities, especially longitudinal studies, which are rare in the Brazilian context and can explain how the changes on the main retirement resources, personal characteristics, or status can influence the well-being.

## Conclusions

This study confirms the importance of social support, economic situation assessment, health assessment, and area of residence for subjective happiness. It suggests that policies and actions that deal with main predictors of happiness in the two environments should be prioritized. Among them, are one’s financial situation, which reinforces the need for long-term financial planning and the diversity of investments facing longevity; social support, which is sought in the community by creation of social living centers; and promotion of health, including physical activities, diet, and healthcare. All of these must be assumed by the government and are present in global efforts of age-friendly cities (Plouffe and Kalache 2010).

Understanding the profile and needs of retirees would mean transforming them in terms of opportunities and reducing weaknesses in both urban and rural areas. Planning of specific services may enable attracting and promoting the return of people who in the past had left the cities and may reduce the overpopulation of these urban centers. Another way to persuade people to stay in their hometown would be the evaluation of public policies and the offering of better life conditions such as high quality healthcare and education services in the environment in which retirees live (Navarro et al. 2015; Ruza et al. 2015).

To prepare cities for active aging (i.e., to turn the city into an age-friendly city) means to prevent problems associated with and respond to needs and preferences related to aging, considering and respecting the decisions of old people and their chosen lifestyle, promoting their inclusion, and mainly, recognizing their contribution in the community (Plouffe and Kalache 2010). These actions must include creation and adaptation involving multiple sectors, such as health, education, safety, work, justice, planning, rural and urban development, housing, transportation, tourism, technologies, culture, and a number of social and individual values (França 2004; Requena 2015).

Happiness is important in all stages of life, and retirement is no different, especially given the contribution already given through the working world and toward the common good. Academia, workers, government, and citizens, individually or collectively, should act about the needs for improvement and possibilities of action in a public and private context.
